# Annealing and Stretching Induced High Energy Storage Properties in All-Organic Composite Dielectric Films

**DOI:** 10.3390/ma11112279

**Published:** 2018-11-14

**Authors:** Yefeng Feng, Cheng Peng, Qihuang Deng, Yandong Li, Jianbing Hu, Qin Wu

**Affiliations:** 1 School of Materials Science and Engineering, Yangtze Normal University, Chongqing 408100, China; feng_ye_feng@126.com (Y.F.); dqhz_a@163.com (Q.D.); andyydlee@gmail.com (Y.L.); hjb2008@163.com (J.H.); 2 Department of Fashion Communication and Media, Jiangxi Institute of Fashion Technology, Nanchang 330201, China; wuqin1028@126.com

**Keywords:** annealing, stretching, energy storage, all-organic, composite

## Abstract

High discharged energy density and charge–discharge efficiency, in combination with high electric breakdown strength, maximum electric displacement and low residual displacement, are very difficult to simultaneously achieve in single-component polymer dielectrics. Plenty of researches have reported polymer based composite dielectrics filled with inorganic fillers, through complex surface modification of inorganic fillers to improve interface compatibility. In this work, a novel strategy of introducing environmentally-friendly biological polyester into fluoropolymer matrix has been presented to prepare all-organic polymer composites with desirable high energy storage properties by solution cast process (followed by annealing or stretching post-treatment), in order to simplify the preparation steps and lower the cost. Fluoropolymer with substantial ferroelectric domains (contributing to high dielectric response) as matrix and poly (3-hydroxybutyrate-*co*-3-hydroxyvalerate) with excellent linear polarization property (resulting in high breakdown strength) as filler were employed. By high-temperature annealing, the size of ferroelectric domains could be improved and interfacial air defects could be removed, leading to elevated high energy storage density and efficiency in composite. By mono-directional stretching, the ferroelectric domains and polyester could be regularly oriented along stretching direction, resulting in desired high energy storage performances as well. Besides, linear dielectric components could contribute to high efficiency from their strong rigidity restrain effect on ferroelectric component. This work might open up the way for a facile fabrication of promising all-organic composite dielectric films with high energy storage properties.

## 1. Introduction

In recent decades, dielectric capacitors have attracted extensive attention in the fields of modern electronic and electrical power systems, ascribed to their fast charge–discharge speed and high energy storage density [1]. Novel dielectric materials with further elevated high discharged energy density and charge–discharge efficiency are highly desired for capacitive applications [2]. It has been widely accepted that the discharged energy density of any material is co-decided by two crucial factors, namely dielectric constant and electric breakdown strength [3]. An improvement of either dielectric constant or breakdown strength can result in an increase of discharged energy density. Linear dielectrics have an energy density that is proportional to the square of breakdown strength and to the first power of dielectric constant [4], such as biaxially oriented polypropylene (BOPP). With regard to non-linear dielectrics such as ferroelectric fluoropolymers, their breakdown strengths contribute more to the achieved energy density than their dielectric constants. That has led to a prevailing trend of employing the materials with high intrinsic breakdown strength to expect a high energy density. 

Polymer dielectrics have been widely investigated as promising candidates due to their ultrahigh breakdown strength, low dielectric loss and light weight [5]. However, several challenges have been found. For instances, the most successful commercial polymer dielectric (namely BOPP) has a rather low dielectric constant [6], while fluoropolymer has an undesired high dielectric loss [7]. These problems have resulted in the development of next-generation polymer dielectrics with new chemistry and unique architectures that are tunable in chemical compositions, flexible in mechanical performances, and stable under high temperature [8]. Recently, the theoretical considerations for rationally designing polymer dielectrics have been proposed [8]. In general, two types of polymer dielectrics (polymers containing dipolar groups in main-chain and side-chain architectures respectively) have been studied [8]. In despite of having achieved sufficient understanding of novel polymer dielectrics with elevated electric properties, there are still some obstacles that can restrict their development. To begin with, the theoretical prediction from the computation is still confined to the investigation of polarizability and bandgap, while the explanation of other key performances such as dielectric loss and breakdown strength demands far more detailed understanding of how applied electric field affects their nanostructures. Next, the state-of-the-art polymer dielectrics need either a very complicated organic synthesis or a highly controlled film-preparation processing, meaning a far distance from a large-scale preparation [5]. Besides, the residual metal catalysts introduced during organic synthesis can be highly harmful to their lifetime. Thus, it is important to develop not only the materials with stable and green synthesis routes, but also the novel processing methods.

So far, blend or composite strategy with the merits of low cost and fast fabrication has been extensively employed in substantial material-related fields [9]. The composites can simultaneously show several advantages from each component of them. In past decades, plenty of promising organic–inorganic composite dielectrics have been reported [10], such as graphene/polymer, barium titanate/polymer and Ni/polymer composites. Although the promising energy storage properties have been obtained, the disadvantages including the poor interfacial compatibility, high leakage conduction, low breakdown strength, and high energy loss have been found in most of the cases. To solve these problems, the surfaces of inorganic fillers have been modified by organic materials to promote the interfacial compatibility or adhesion [11]. However, both of the high-cost and time-consuming shortcomings have been found.

Nowadays, all-organic composite dielectrics made from polymeric components have incurred wide attention and research interest in the field of high-density energy storage [12]. Different from organic–inorganic composite dielectrics discussed above, the all-organic composite dielectrics consisting of two or more polymeric components can possess a higher interfacial compatibility between any two components to avoid undesired high interface-induced leakage conduction, low breakdown strength, and low charge–discharge efficiency [13]. Moreover, the interaction (such as rigidity limit effect) between two varied polymeric constituents in all-organic composite dielectrics is highly tunable, resulting in controllable dielectric and energy storage performances [14]. In this work, we have elaborately designed and prepared a series of all-organic composite films with the promising high energy density and efficiency, based on a solution cast process followed by post-treatments (including high-temperature annealing and mono-directional stretching). This work has been designed based on the concept of ‘all-organic composite material’, the effect of rigidity restriction of linear dielectric material on the ferroelectric material, and the mature polymer post-processing methods of annealing and stretching. This work aims at providing a novel strategy for preparing promising composite dielectric materials, through building all-organic composites containing ferroelectric and linearly dielectric components as well as utilizing material post-treatments to further improve the energy storage performances of composites. Firstly, several PVDF based co-polymers, including poly(vinylidene fluoride-*co*-trifluoroethylene-*co*-chlorotrifluoroethylene) (P(VDF-TrFE-CTFE)) and poly(methyl methacrylate)-grafted P(VDF-TrFE-CTFE) (labeled as P(VDF-TrFE-CTFE)-g-PMMA), have been employed as polymer matrices, due to a large number of ferroelectric domains (contributing to high dielectric response) and a small size of those domains (contributing to low residual displacement and energy loss) [15]. Secondly, a green environmentally-friendly biological polyester, namely poly(3-hydroxybutyrate-*co*-3-hydroxyvalerate) (PHBV, called ‘green plastic’), has been utilized as polymer filler, ascribed to its excellent linear polarization property (to improve the breakdown strength) and strong rigidity restriction effect (to further tailor the size of ferroelectric domains in matrices) [16]. Finally, the mature post-treatment methods (annealing and stretching) have been adopted to process the shape-formed composite films to further improve the maximum electric displacement and breakdown strength. The desirable high energy density (9.4 J·cm^−3^) and charge–discharge efficiency (70%) have been achieved in the stretched composite film. This work might open up the way for a large-scale preparation of high-performance all-organic composite dielectric films.

## 2. Materials and Methods

Poly(vinylidene fluoride-*co*-trifluoroethylene-*co*-chlorotrifluoroethylene) (P(VDF-TrFE-CTFE), with TrFE units of 15 mol % and CTFE units of 5 mol %, abbr. 80-15-5, 99%, AR grade) and poly(vinylidene fluoride-trifluoroethylene-chlorotrifluoroethylene)-g-poly(methyl methacrylate) (P(VDF-TrFE-CTFE)-g-PMMA, with VDF units of 80 mol % and TrFE units of 15 mol % in main chains, as well as with PMMA side chains of 20 wt % and 30 wt % respectively in the entire macromolecule, abbr. 80-15-5-g-PMMA-20 and 80-15-5-g-PMMA-30 respectively, 99%, AR grade) were bought from Solvay. Poly(3-hydroxybutyrate-*co*-3-hydroxyvalerate) (PHBV, with 3-hydroxyvalerate units of 2 mol %, 99%, AR grade) was purchased from Shanghai Gujie Trading Co., Ltd. (Shanghai, China). Poly(methyl methacrylate) (PMMA, 99%, AR grade) and N,N-dimethylformamide (DMF, 99.5%, AR grade) were obtained from Aladdin. All materials were used as received, without any treatment.

The preparation of all-organic composite dielectric films was clarified as follows: five composite systems, namely 20 wt % PHBV filled 80-15-5 based composite (80-15-5/PHBV-20), 10 wt % PHBV filled 80-15-5-g-PMMA-20 based composite (80-15-5-g-PMMA-20/PHBV-10), 5 wt % PHBV filled 80-15-5-g-PMMA-30 based composite (80-15-5-g-PMMA-30/PHBV-5), 10 wt % PHBV filled 80-15-5-g-PMMA-30 based composite (80-15-5-g-PMMA-30/PHBV-10) and 10 wt % PHBV filled PMMA based composite (PMMA/PHBV-10), were prepared into films by a solution cast method [17] from a mixture solution (3 wt % of total concentration for two polymer components) containing the above designed weight fractions of PHBV, with DMF as the solvent, at 70 °C, on neat glass slides. Besides of the composite films mentioned above, several neat polymer films—including 80-15-5, PHBV, and PMMA films—were prepared through a solution cast process as well in the similar way to composite films. In this work, some annealed or stretched film samples, including annealed 80-15-5 (abbr. 80-15-5(A)), annealed 80-15-5/PHBV-20 (80-15-5/PHBV-20(A)), stretched 80-15-5-g-PMMA-30/PHBV-5 (80-15-5-g-PMMA-30/PHBV-5(S)), stretched 80-15-5-g-PMMA-30/PHBV-10 (80-15-5-g-PMMA-30/PHBV-10(S)), stretched PMMA/PHBV-10 (PMMA/PHBV-10(S)) and stretched PMMA (PMMA(S)), were fabricated as well by annealing or stretching post-treatment (after the complete evaporation of DMF from the samples).

Annealing was conducted at 180 °C in an oven for 6 h, followed by slowly cooling the samples (with their glass substrates) to room temperature. It aimed at removing the air defects and increasing the size of crystalline grains in the samples. After the annealing, the film samples (with a thickness of ca. 18 μm) were fully peeled off from glass slides, followed by sputtering with Au on both surfaces of the films as electrodes for electric property measurements. Moreover, the stretching with a draw ratio of 2:1 was carried out for film samples (peeled off from glass slides, without substrates) at 100 °C, by immobilizing one end of the film and continually applying a constant tension stress at the other end of the film (a constant tension stress was ascertained by using a clamp and a heavy (ca. 100 g)) in the vertical direction. After the stretching, the film samples with a thickness of ca. 18 μm were obtained, followed by sputtering with Au on their both surfaces as electrodes for electric performance tests. To verify the full drying of all as-prepared films before property tests, thermogravimetric analysis (TGA) result of neat PHBV film was obtained in Figure S1.

Proton nuclear magnetic resonance (^1^H NMR) spectra were achieved by a Bruker (Advance III) 400 MHz spectrometer with tetramethylsilane as the internal standard (deuterated acetone and deuterated furanidine as the solvents for PVDF based co-polymers and PHBV, respectively). Mono-polar and bi-polar electrical hysteresis loops (*D*-*E* loops) were obtained by a ferroelectric analyzer (Premiere II, Radiant Technologies). Direct-current electric field was applied to test mono-polar *D*-*E* loops, while alternative-current one to measure bi-polar *D*-*E* loops. The basic testing conditions included the applied field with a triangular waveform, frequency of 10 Hz and room temperature. Dielectric properties, including dielectric constant and loss results (at room temperature), were achieved by a HP4284A LCR meter under the frequencies at 100 Hz–1 MHz with a bias voltage of 1 V. Au electrodes were deposited on both surfaces of all films through a JEOL JFC-1600 auto fine coater (Tokyo, Japan) before all the electric performance tests.

## 3. Results and Discussion

### 3.1. Characterization of Original Materials and Study of Dielectric Property

**Figure 1:**
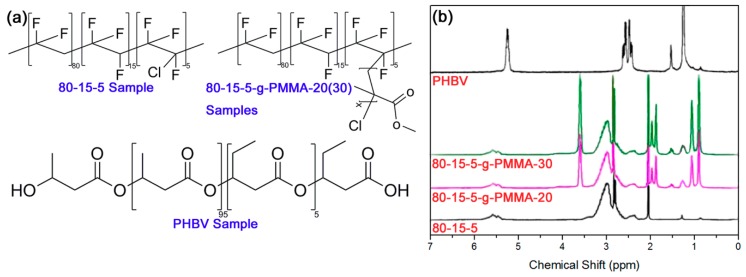

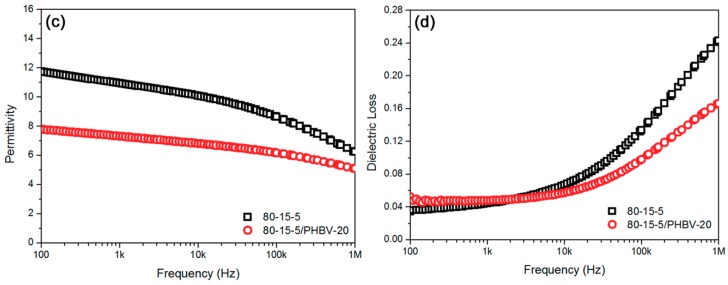
(**a**) Schemed diagrams showed the molecular structures of four main original materials, (**b**) 1H NMR results confirmed the chemical compositions of the four materials; (**c**) Permittivity results showed a reduction of the permittivity for composite sample in comparison to neat 80-15-5 sample, ascribed to a reduction of the size of ferroelectric domains in 80-15-5 component from dilution effect of PHBV component; and (**d**) loss results showed a decrease of loss for composite at high frequency, compared with neat 80-15-5, ascribed to low dielectric response in PHBV component as well as reduction of inner friction during dipoles reversal in 80-15-5 component.

The schemed diagrams of the molecular structures of four original materials were shown in Figure 1a. Figure 1b exhibited their ^1^H NMR results. Based on the substantial reports [18,19], the declared chemical compositions for them in Materials and Methods section could be confirmed. The permittivity and dielectric loss for 80-15-5 and 80-15-5/PHBV-20 samples were obtained as a function of frequency in Figure 1c and Figure 1d, respectively. In comparison to 80-15-5, an introduction of 20 wt % of PHBV would result in a reduction of permittivity, attributed to the dilution effect of PHBV with lower polarity [18]. Based on Figure 1d, dielectric loss of composite was slightly higher than 80-15-5 at lower frequencies, due to the interphase formed between two components (interface-induced leakage-conduction loss) in 80-15-5/PHBV-20 [19]. The composite exhibited a lower loss than 80-15-5 at the frequencies between 10 kHz and 1 MHz. That might be clarified by a lower dielectric response and thus lower loss for low-polarity PHBV in composite than 80-15-5 with high-polarity ferroelectric domains [20]. Meanwhile, PHBV would dilute the crystalline grains of 80-15-5, leading to a decrease of inner-friction-induced loss during the reversal of dipoles in 80-15-5 [21].

### 3.2. Mono-Polar and Bi-Polar D-E Loops of All As-Prepared Film Samples

**Figure 2:**
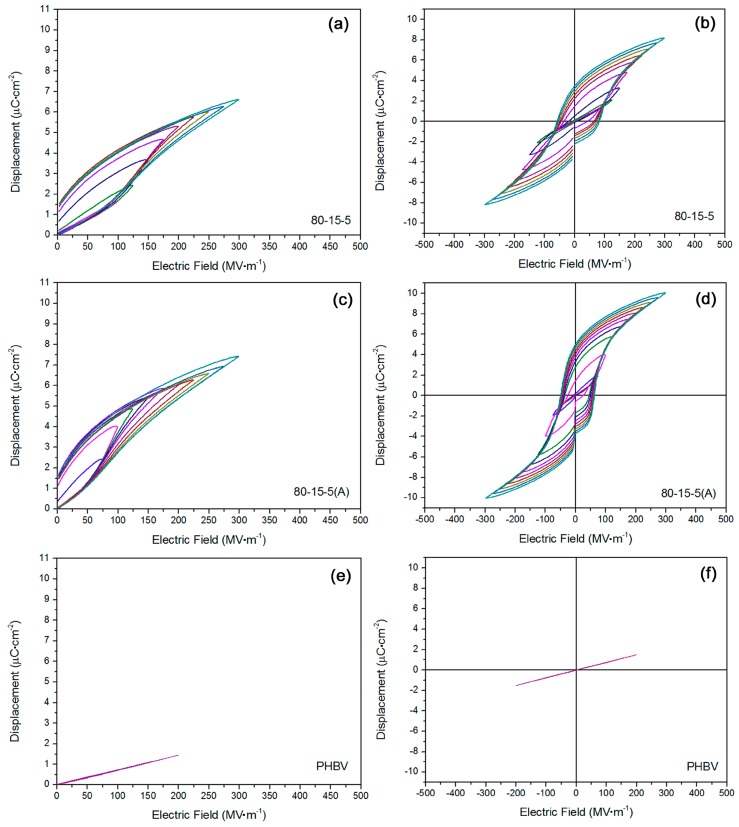
(**a**,**b**) *D*-*E* loops showed the relaxor ferroelectric nature in 80-15-5 sample; (**c**,**d**) *D*-*E* loops exhibited the annealing induced high electric displacement and low coercive electric field for 80-15-5 sample; and (**e**,**f**) *D*-*E* loops confirmed the linear dielectric feature for neat PHBV sample.

Mono-polar and bi-polar *D*-*E* loops for 80-15-5, PHBV and annealed 80-15-5 samples were obtained in Figure 2. Based on Figure 2a,b, the relaxor ferroelectric property was verified in 80-15-5, suggesting a superiority for an energy storage to ferroelectric polymers thanks to the large-volume CTFE units in 80-15-5 [22]. In Figure 2a, with an increase of applied field, the maximum displacement of 80-15-5 was gradually elevated. Fat hysteresis loops could suggest a higher energy loss. In Figure 2b, the corresponding bi-polar *D*-*E* loops were exhibited.

Annealing processing has been widely used to promote the high-field dielectric properties and the quality of hysteresis loops for ferroelectric polymers [23]. In Figure 2c,d, *D*-*E* loops for annealed 80-15-5 were displayed. By comparison, annealing would not elevate the breakdown strength, but it would result in the slim *D*-*E* loops (in comparison with unannealed 80-15-5). Based on Figure 2a,c, the highest polarization increased from 6.6 µC cm^−2^ to 7.4 µC cm^−2^ at breakdown strength, after annealing. That originated from the annealing-induced higher degree of crystallinity and larger crystalline grain size in 80-15-5 [24]. In Figure 2e,f, *D*-*E* loops of PHBV were exhibited. The linear dielectric nature could be confirmed. As expected, both of the residual displacement and coercive field at 200 MV m^−1^ were rather close to zero, suggesting a low energy loss. According to bi-polar hysteresis loops in Figure 2f, linear dielectric nature was further verified.

**Figure 3:**
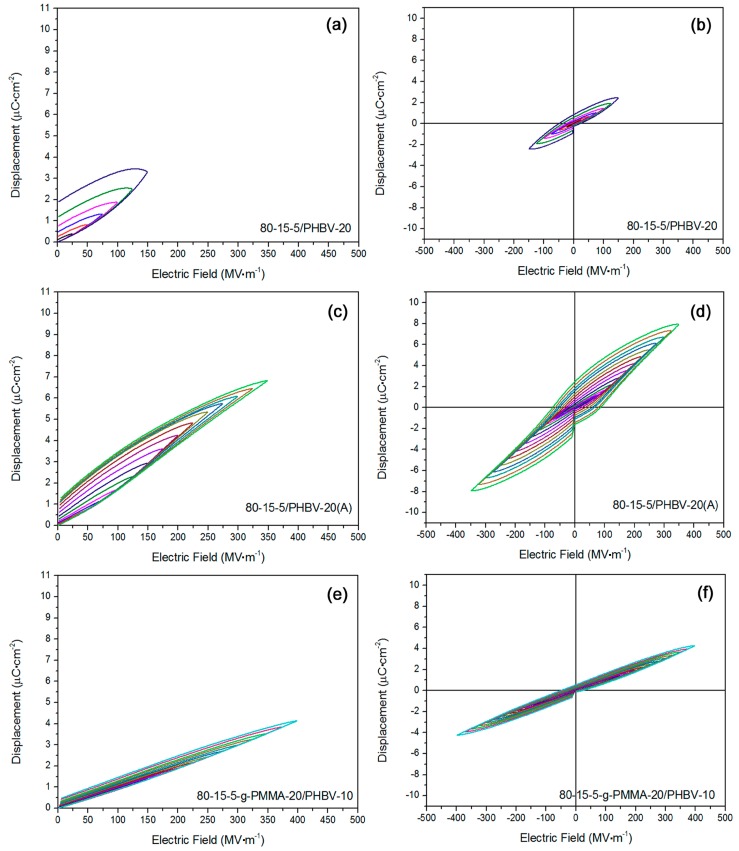
(**a**,**b**) *D*-*E* loops showed high leakage conduction in 80-15-5/PHBV-20 sample; (**c**,**d**) *D*-*E* loops exhibited relaxor ferroelectric nature in annealed 80-15-5/PHBV-20 sample with high breakdown strength and large electric displacement; and (**e**,**f**) *D*-*E* loops verified linear dielectric nature in 80-15-5-g-PMMA-20/PHBV-10 sample with high breakdown strength and low residual polarization.

In Figure 3a,b, the hysteresis loops for 80-15-5/PHBV-20 were exhibited. *D*-*E* loops became very fat, suggesting a high leakage current. Mono-polar hysteresis loop at its breakdown strength in Figure 3a suggested a high leakage conduction. The highest displacement at the breakdown strength was far lower than 80-15-5, ascribed to an introduction of low-polarity PHBV and the low-breakdown-strength induced unsaturated polarization [25]. Similarly, annealing was adopted again to treat 80-15-5/PHBV-20 sample. In Figure 3c,d, *D*-*E* loops for annealed 80-15-5/PHBV-20 were shown. Compared with the unannealed counterpart, this annealed composite regained a relaxor ferroelectric property. First of all, the breakdown strength improved to 350 MV m^−1^ (by 133%), attributed to annealing-induced low concentration of interfacial defects and high mechanical modulus [26]. Besides, the maximum displacement (ca. 7–8 µC·cm^−2^) for annealed sample at its breakdown strength significantly improved. Next, based on Figure 3a,c, annealing led to a reduced residual displacement.

In Figure 3e,f, *D*-*E* loops for 80-15-5-g-PMMA-20/PHBV-10 were obtained. Herein, 80-15-5-g-PMMA-20 component had a linear-like dielectric property, and PHBV component had a nice linear dielectric feature. Based on the results in Figure 3e,f, a highly linear dielectric property was confirmed for 80-15-5-g-PMMA-20/PHBV-10. Its breakdown strength was ca. 400 MV m^−1^, which was higher than the annealed 80-15-5/PHBV-20. The maximum displacement at breakdown strength for 80-15-5-g-PMMA-20/PHBV-10 was ca. 4 µC·cm^−2^, which was much lower than corresponding results in Figure 3c,d. That was ascribed to the rigidity restriction effect of PMMA on the reversal of dipoles in 80-15-5 [27], for 80-15-5-g-PMMA-20 matrix in the composite.

**Figure 4:**
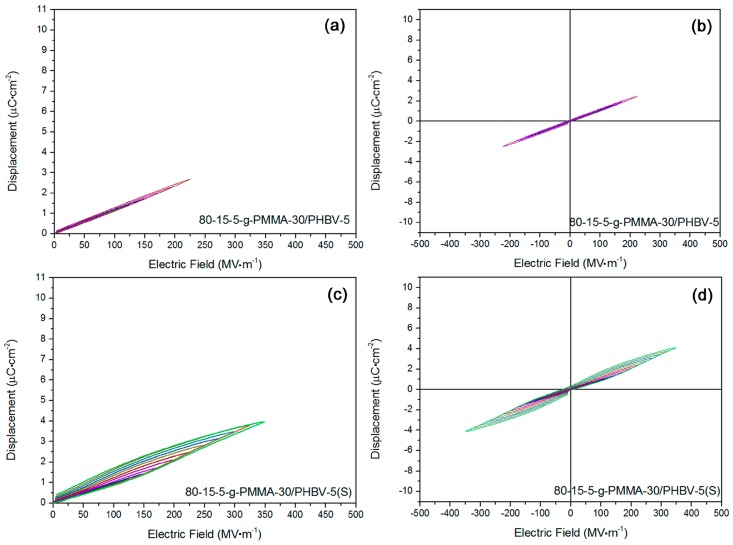

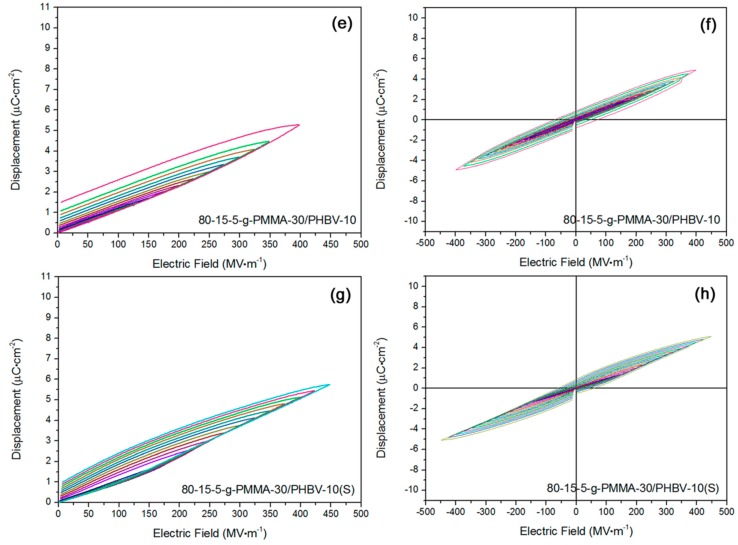
(**a**,**b**) *D*-*E* loops showed fine linear dielectric nature in 80-15-5-g-PMMA-30/PHBV-5 sample; (**c**,**d**) *D*-*E* loops exhibited anti-ferroelectric feature in stretched 80-15-5-g-PMMA-30/PHBV-5 sample; (**e**,**f**) *D*-*E* loops showed degraded linear dielectric behavior in 80-15-5-g-PMMA-30/PHBV-10 sample; and (**g**,**h**) they showed relaxor ferroelectric trait in stretched 80-15-5-g-PMMA-30/PHBV-10 sample.

Another 80-15-5-g-PMMA-30/PHBV-5 sample was studied as well. In Figure 4a,b, its *D*-*E* loops were shown. Linear dielectric characteristic was found in it. The highest polarization at breakdown strength was ca. 2.6 µC·cm^−2^. Such a low displacement was ascribed to a reduction of breakdown strength and thus an insufficient polarization. To improve the energy storage properties, stretching treatment was conducted, and *D*-*E* loops were obtained in Figure 4c,d. Similarly, anti-ferroelectric behavior was confirmed. Stretching-induced orientation for small-sized crystalline grains in 80-15-5 main chains should be responsible for the anti-ferroelectric property [28]. Stretching would promote the degree of crystallinity and get rid of interfacial air defects, possibly resulting in an improved Young’s modulus (contributing to elevated breakdown strength) [29]. To clarify the influence of PHBV on high-field dielectric property, 80-15-5-g-PMMA-30/PHBV-10 sample was investigated and its D-E loops were displayed in Figure 4e,f. A high leakage conduction was found (see fat loops). That was ascribed to a proposed microscopic phase separation between two components from high concentration of PHBV [30]. Stretching was also carried out for 80-15-5-g-PMMA-30/PHBV-10, and its *D*-*E* loops were exhibited in Figure 4g,h. Interestingly, after stretching, the sample showed a relaxor ferroelectric property. Through that stretching, 80-15-5-g-PMMA-30/PHBV-10 would obtain a higher breakdown strength, attributed to stretching-induced elevation of Young’s modulus [31].

**Figure 5:**
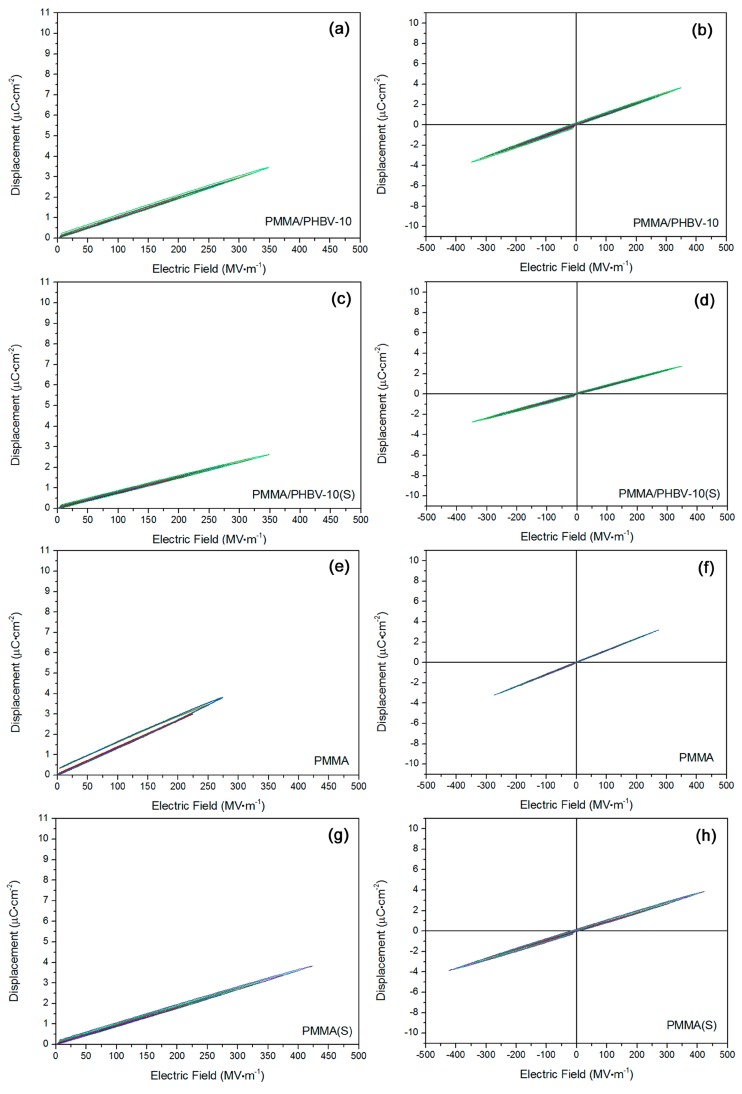
Fine linear dielectric nature was confirmed based on *D*-*E* loops results for (**a**,**b**) PMMA/PHBV-10 sample; (**c**,**d**) stretched PMMA/PHBV-10 sample, (**e**,**f**) neat PMMA sample; and (**g**,**h**) stretched PMMA sample.

In Figure 5a,b, *D*-*E* loops for PMMA/PHBV-10 sample were obtained. The composite had a nice linear polarization property, ascribed to its all-linear dielectric components. Both of the residual polarization and coercive field at breakdown strength could be deemed as ca. 0. Based on Figure 5c,d, *D*-*E* loops for stretched PMMA/PHBV-10 were exhibited. After a stretching, the fine linear dielectric property was still maintained. Based on Figure 5e,f, *D*-*E* loops for PMMA were displayed. This sample showed a good linear dielectric property. Similarly, stretching was operated for PMMA, and the corresponding results were shown in Figure 5g,h. As expected, the linear polarization property was still kept. Fortunately, the breakdown strength was improved, due to the hypothetical stretching-induced local crystallization behavior in PMMA [32].

### 3.3. Energy Storage Properties of All As-Prepared Film Samples

**Figure 6:**
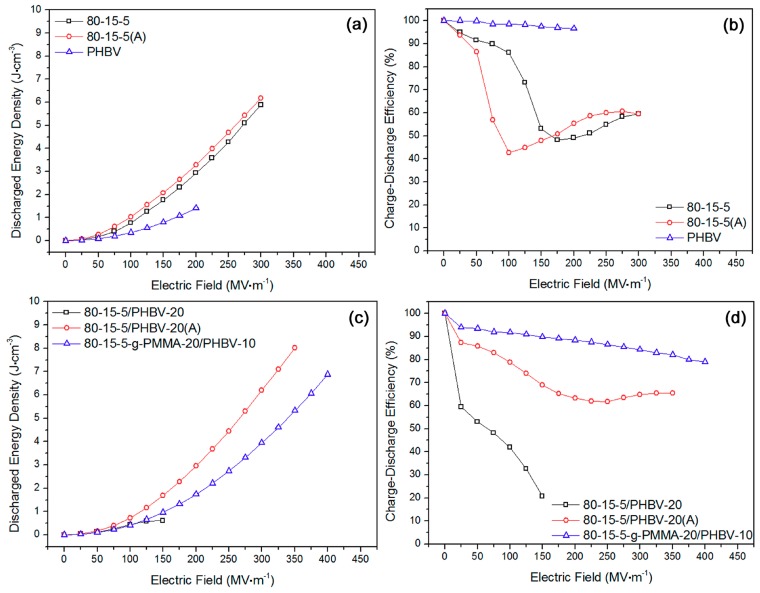
No significant elevation of energy storage properties after the annealing was verified for (**a**,**b**) 80-15-5 sample, and notable improvement of energy storage performances after annealing was confirmed for (**c**,**d**) 80-15-5/PHBV-20 sample.

**Figure 7:**
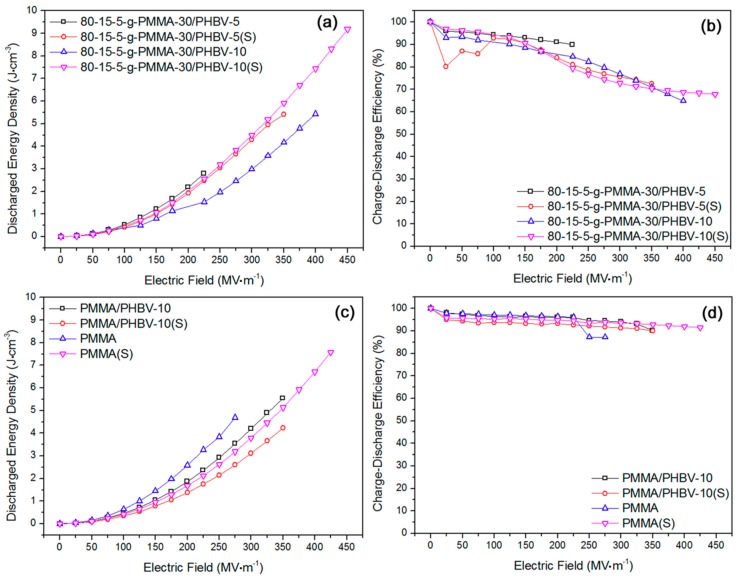
(**a**) High energy density was achieved in stretched 80-15-5-g-PMMA-30/PHBV-10 sample; (**b**) stretching induced reduction of charge–discharge efficiency was found for the two systems on the whole; (**c**) high energy density was obtained in stretched PMMA sample; and (**d**) insignificant influence of stretching on efficiency was verified for the two systems in wide applied field range.

Based on those mono-polar *D*-*E* loops, both of the discharged energy density and charge–discharge efficiency of some samples were displayed in Figure 6 and Figure 7. According to Figure 6a, with an increase of field, the energy density for 80-15-5, annealed 80-15-5 and PHBV gradually elevated. The energy density of 80-15-5 was improved much faster than PHBV. After the annealing, the energy density of 80-15-5 slightly improved, at the same field. In Figure 6b, corresponding efficiency results were shown. The efficiency of PHBV slowly reduced, as the applied field was increased. Interestingly, the efficiency of 80-15-5 and annealed 80-15-5 samples reduced firstly and then increased with an increase of electric field, ascribed to the difference of *D*-*E* loops at low and high applied fields for relaxor ferroelectric 80-15-5 material (see Figure 2a,b). In detail, when the applied field was relatively low, an increase of electric field could lead to the fatter and fatter *D*-*E* loops due to weak-polarization induced complex orientation states of ferroelectric domains (suggesting the increase of inner friction force during the relaxation of dipoles) under low fields. However, when the applied field was over some critical field and relatively high, an increase of applied electric field could result in the slimmer and slimmer *D*-*E* loops ascribed to strong-polarization induced simple orientation states of domains (suggesting the reduction of inner friction force during dipole relaxation) under high fields. In Figure 6c, annealing resulted in a promotion of energy density for 80-15-5/PHBV-20. The breakdown strength was sharply improved by 133%. The maximum energy density at breakdown strength was elevated by 1233% (from 0.6 J·cm^−3^ to 8 J·cm^−3^). Based on Figure 6d, the corresponding efficiency results were displayed. Annealing would remarkably improve the efficiency at the same field, for 80-15-5/PHBV-20. Moreover, annealing resulted in an elevation of breakdown strength. Similarly, the efficiency of annealed 80-15-5/PHBV-20 sample decreased firstly and then improved, attributed to the 80-15-5 component (specific reason for the changing trend was stated above) in that composite. Because of the lower breakdown strength (150 MV·m^−1^), the increasing trend of the efficiency of 80-15-5/PHBV-20 sample at high fields could not be observed.

According to Figure 7a, the stretching contributed to an increase of breakdown strength. Stretched 80-15-5-g-PMMA-30/PHBV-5 had an elevated maximum energy density in comparison to the unstretched counterpart. However, with regard to 80-15-5-g-PMMA-30/PHBV-10, the stretching could obviously improve the energy density. In Figure 7b, the corresponding efficiency results were exhibited. On the whole, stretching could not significantly improve the efficiency. That might be rooted in the high concentration of PMMA side chains. Before that stretching, a low energy loss has been achieved based on a strong rigidity restriction effect [33]. Based on Figure 7c, stretching led to a negative effect on the energy density of PMMA/PHBV-10. In Figure 7d, the efficiency results were displayed. A high efficiency (over 90%) was gained for those samples.

### 3.4. Comparison Study on All the As-Prepared Samples

The important parameters achieved in all samples were summarized in Table 1. The maximum displacement and residual displacement were achieved based on mono-polar *D*-*E* loops. All data were achieved at the respective breakdown strength of each sample. By contrast, three samples (see bold fonts in ‘Samples’ column) should be emphasized. 80-15-5-g-PMMA-20/PHBV-10 had a high efficiency (84%) and low residual displacement (0.4 µC·cm^−2^). Annealed 80-15-5/PHBV-20 had a high energy density (8.1 J·cm^−3^) and maximum electric displacement (6.8 µC·cm^−2^). Stretched 80-15-5-g-PMMA-30/PHBV-10 had a high energy density (9.4 J·cm^−3^) and breakdown strength (450 MV·m^−1^). To conclude, the proper total concentration and ratio for both linear dielectric components (PHBV and PMMA) should be helpful to a high efficiency. More importantly, both of annealing and stretching treatments could greatly contribute to an improvement of energy density.

**Figure 8:**
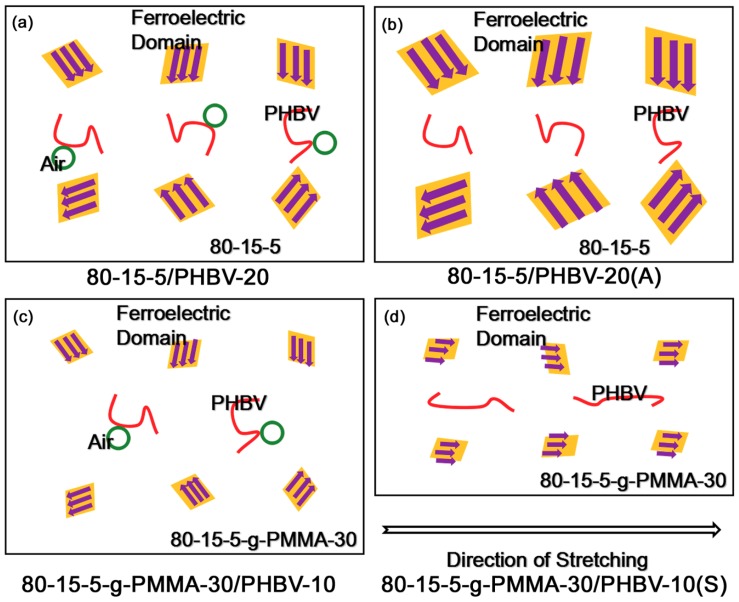
Schematic diagrams of (**a**) 80-15-5/PHBV-20 sample with randomly oriented middle ferroelectric domains and with interface air defects; (**b**) annealed 80-15-5/PHBV-20 sample with randomly oriented large ferroelectric domains and with high interface adhesion; (**c**) 80-15-5-g-PMMA-30/PHBV-10 sample with randomly oriented small ferroelectric domains and with air defects; and (**d**) stretched 80-15-5-g-PMMA-30/PHBV-10 sample with regularly oriented small ferroelectric domains and PHBV as well as with high interface adhesion.

In Figure 8, the schemed diagrams of four samples (80-15-5/PHBV-20, 80-15-5/PHBV-20(A), 80-15-5-g-PMMA-30/PHBV-10, and 80-15-5-g-PMMA-30/PHBV-10(S)) were exhibited. Based on Figure 8a, the 80-15-5 component in 80-15-5/PHBV-20 should have the ferroelectric domains with a medium size and random orientation, ascribed to the rigidity limit effect of PHBV. Because of the simple physical blend, air defects at interfacial zone might exist. In Figure 8b, annealed 80-15-5/PHBV-20 should have the ferroelectric domains with an increased size, compared with Figure 8a, due to an improvement of the size of crystalline grains from annealing induced better growth of the crystalline grains in 80-15-5. That contributed to an elevation of the maximum displacement. Air defects might be removed by that annealing treatment [34]. That was helpful to an improvement of breakdown strength.

In Figure 8c, 80-15-5-g-PMMA-30/PHBV-10 should have the ferroelectric domains with a reduced size (compared with Figure 8a), attributed to the further enhanced rigidity limit effect of high concentration of PMMA. Air defects might be formed. However, in Figure 8d, the stretched counterpart should possess the ferroelectric domains with a regular orientation along the direction of stretching [35]. The regular orientation could bring about an increase of the maximum displacement. Moreover, stretching might induce a regular orientation of PHBV along the stretching direction, and it might remove air defects [36]. That improved the breakdown strength. The residual polarization was reduced, due to an enhanced rigidity restraint effect from a shortened molecular distance between PHBV and 80-15-5-g-PMMA-30 [37].

## 4. Conclusions

In this work, a facile and practical route for fabricating all-organic polymer composite films with desirable high energy storage properties has been presented. To avoid complicated surface modification of inorganic filler to improve its interface compatibility with polymeric matrix, an organic polymeric filler (a kind of green and environmentally-friendly biological polyester, PHBV) has been introduced into PVDF based polymer matrix to build the composites at a reduced cost. Nine composite systems totally have been fabricated, based on different classes of polymer matrices, weight concentrations of PHBV filler, and post-treatments of composites. By detailed investigation on *D*-*E* loops and energy storage properties of all samples, three promising composites have been found, namely 80-15-5-g-PMMA-20/PHBV-10, annealed 80-15-5/PHBV-20, and stretched 80-15-5-g-PMMA-30/PHBV-10 composites. Both of the proper total concentration and feeding ratio for PHBV and PMMA with excellent linear dielectric properties have been found to greatly contribute to a favorable high charge–discharge efficiency from dual-linear-dielectrics induced strong rigidity limit effect and ultra-low residual displacement. Moreover, two very important post-processing methods (high-temperature annealing and mono-directional stretching) have been found to result in the desired high discharged energy density and energy efficiency from significant improvement of the maximum displacement and breakdown strength. Annealing treatment has been used to improve the size of ferroelectric domains (increasing the maximum displacement) and remove the interfacial air defects (elevating the breakdown strength), while stretching has been applied to induce a regular orientation of ferroelectric domains (improving the displacement), and PHBV component (increasing the breakdown strength) along the stretching direction. Therefore, the desired high energy density and efficiency have been achieved in annealed and stretched all-organic polymer composites filled with the environmentally-friendly linear dielectric. 

## Figures and Tables

**Table 1:** Important parameters for all the samples at their own breakdown strengths.

Samples	Breakdown Strength (MV·m^−1^)	Maximum Displacement (µC·cm^−2^)	Residual Displacement (µC·cm^−2^)	Energy Density (J·cm^−3^)	Energy Efficiency (%)
80-15-5	300	6.7	1.4	6.0	60
80-15-5(A)	300	7.5	1.5	6.4	60
PHBV	200	1.5	~0	1.5	97
80-15-5/PHBV-20	150	3.4	1.9	0.6	20
**80-15-5/PHBV-20(A)**	**350**	*6.8*	1.1	*8.1*	**68**
**80-15-5-g-PMMA-20/PHBV-10**	**400**	**4.1**	*0.4*	**7.1**	*84*
80-15-5-g-PMMA-30/PHBV-5	225	2.7	~0	2.9	92
80-15-5-g-PMMA-30/PHBV-5(S)	350	4.0	0.4	5.5	75
80-15-5-g-PMMA-30/PHBV-10	400	5.4	1.4	5.6	67
**80-15-5-g-PMMA-30/PHBV-10(S)**	*450*	**5.8**	**0.9**	*9.4*	**70**
PMMA/PHBV-10	350	3.6	~0	5.9	92
PMMA/PHBV-10(S)	350	2.8	~0	4.4	92
PMMA	275	3.9	~0	4.8	88
PMMA(S)	425	3.9	~0	7.8	94

## Supplementary Materials

The following are available online at http://www.mdpi.com/1996-1944/11/11/2279/s1, Figure S1: TGA result confirmed the full drying of as-prepared PHBV film; Figure S2: Cross-section SEM result of 80-15-5/PHBV-20 film confirmed high interface compatibility; Figure S3: XRD results of 80-15-5 and 80-15-5/PHBV-20 films confirmed rigidity limit effect of PHBV.
