# Supplementary material for: Annealing and Stretching Induced High Energy Storage Properties in All-Organic Composite Dielectric Films
Source: Materials (Basel). 2018 Nov 14;11(11):2279. doi: 10.3390/ma11112279 (PMC6266785; doi:10.3390/ma11112279)
Supplement: Supplementary file 1 [file materials-11-02279-s001.pdf]

Supporting Information for Publication

Annealing and Stretching Induced High Energy Storage Properties in All-Organic Composite Dielectric Films

Yefeng Feng, Cheng Peng*, Qihuang Deng, Yandong Li, Jianbing Hu, Qin Wu

Thermogravimetric analysis (TGA) of neat PHBV film was carried out on a NJKHTG-1, and the result was exhibited in Figure S1. The decomposition temperature of PHBV was detected to be ca. 250 °C. No decrease of the mass of the sample at ca. 150 °C (boiling point of DMF is 152.8 °C), suggesting the full drying of as-prepared PHBV film.

The cross-section SEM result of 80-15-5/PHBV-20 composite film was achieved by a JEOL JSM-6700F, as shown in Figure S2. No obvious phase separation was observed, suggesting high interface compatibility in the film.

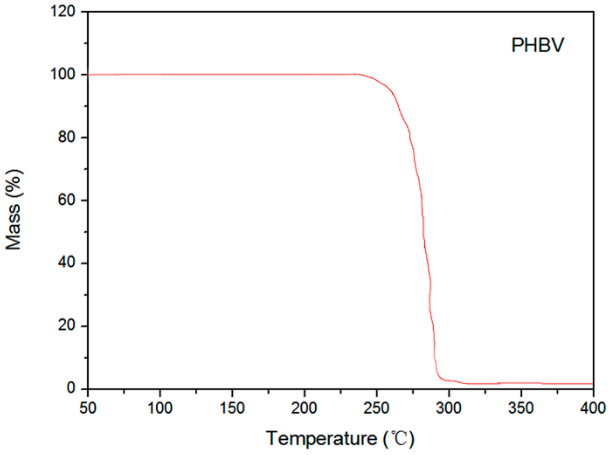

Figure S1. TGA result confirmed the full drying of as-prepared PHBV film.

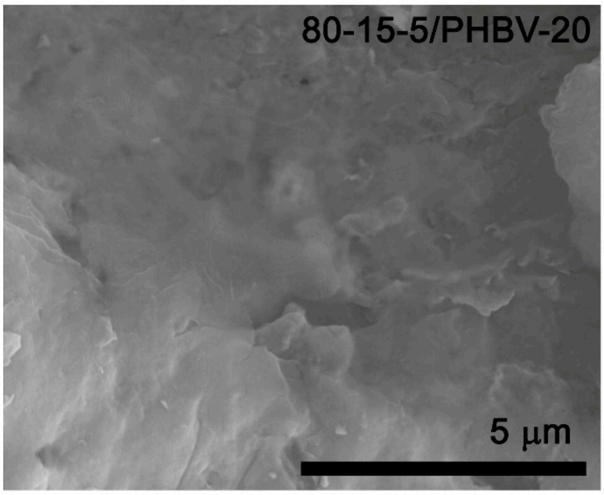

Figure S2. Cross-section SEM result of8.
